# Expression of Ki-67 in Invasive Breast Carcinoma and Its Correlation With Different Clinicopathological Features

**DOI:** 10.7759/cureus.69820

**Published:** 2024-09-20

**Authors:** Upasana Sandilya, Mamatha K

**Affiliations:** 1 Pathology, Bijapur Lingayat District Education (BLDE) (Deemed to be University) Shri BM Patil Medical College Hospital and Research Centre, Vijayapura, IND

**Keywords:** histological grading, immunohistochemistry staining, invasive breast cancer, ki-67, prognosis

## Abstract

Introduction

Worldwide, breast cancer is still the most common cancer that affects women. Breast cancer prognosis is based on a number of clinical and pathological indicators. Further features are needed to predict early metastasis and prognosis of patients with breast carcinoma, as the current pathological characteristics, such as tumor differentiation, vascular infiltration, and TNM (tumor, node, and metastasis) staging, cannot fully describe the early metastatic biological behavior in breast carcinoma. High Ki-67 expression is seen to be associated with worse survival in cancer patients. Hence, this study was conducted to study the utility of Ki-67 as a prognostic marker in invasive breast carcinoma and its association with known clinicopathological factors.

Methodology

The study was a hospital-based cross-sectional study carried out in the Histopathology Section of the Department of Pathology, Shri BM Patil Medical College Hospital and Research Centre, Bijapur Lingayat District Education (BLDE) (Deemed to be University), Vijayapura. The study was conducted between September 1, 2022, and April 30, 2024. The study population consisted of 55 cases of mastectomy specimens of primary breast cancer admitted to our hospital. Data regarding the patient's age, tumor size, histological type, histological grade, lymph node status, and vascular invasion were noted from medical records. Immunohistochemical staining for estrogen receptor (ER), progesterone receptor (PR), human epidermal growth factor receptor 2/neu (HER2/neu) proto-oncogene, and Ki-67 markers was performed according to standard protocol. The relationship of Ki-67 with these clinicopathological parameters was analyzed statistically.

Results

In the current study, high-grade Ki-67 nuclear positivity was seen in 30 cases out of 55 cases, and low-grade Ki-67 was seen in the remaining 25 cases. The association of Ki-67 with tumor size and histological grade showed statistical significance with a p-value of less than 0.05 and 0.001, respectively. However, no statistical significance was seen with lymph node status, vascular invasion, estrogen receptor (ER), progesterone receptor (PR), and human epidermal growth factor receptor 2/neu (HER2/neu) proto-oncogene status with p-values greater than 0.05.

Conclusion

The expression of Ki-67 was statistically significant with histological grading and tumor size in our study. Immunohistochemical determination of the Ki-67 proliferation index should be performed in routine cases of breast cancer to obtain clinically useful information on tumor aggressiveness as reflected in their proliferative rate. Hence, Ki-67 can be used as a predictive and prognostic marker in managing breast cancer patients.

## Introduction

Breast cancer is the most common cancer that affects women's health [1]. It is the most frequent cancer that kills women globally with over a million incidences being diagnosed annually [1-3]. Data from the Global Cancer Statistics 2022 showed that among women worldwide, carcinoma of the breast has the highest incidence. Deaths from malignant breast tumors account for 6.9% of all malignant tumor-related mortality and rank sixth in the death rate due to malignant tumors and their tendency to rapidly increase [4].

The prognosis of breast cancer is based on a number of clinical and pathological indicators. These include the status of lymph nodes and tumor size, type, and grade, all of which affect the result [5]. Nevertheless, other histopathological features are needed to predict early metastasis and the prognosis of breast carcinoma patients, as the current pathological characteristics, such as tumor differentiation, vascular infiltration, and TNM (tumor, node, and metastasis) staging, cannot fully reflect the early metastatic biological behavior in carcinoma breast [6]. Researching new breast cancer prognostic indicators has gained traction in recent years [7]. Human epidermal growth factor receptor 2/neu (HER2/neu) expression and hormone receptor status, including estrogen receptor (ER) and progesterone receptor (PR), are widely employed as biomarkers for breast cancer and are analyzed using immunohistochemistry (IHC) [3].These biomarkers are used as prognostic indicators to predict response to hormonal and chemotherapeutic treatments and in choosing adjuvant therapy for breast cancer. In addition to estrogen receptor (ER), progesterone receptor (PR), and human epidermal growth factor receptor 2/neu (HER2/neu), a number of additional markers have been thoroughly investigated to determine their association with the clinicopathological features of breast cancer as well as their function in treatment selection and outcome modification. One such marker is Ki-67 [5]. One of the most significant prognostic indicators is the proliferation of the tumor. Nuclear protein Ki-67 is expressed during cell division and indicates the percentage of cell division [8]. It is a cancer biomarker that measures the percentage of cells in a tissue sample that are dividing. A higher Ki-67 index indicates a faster rate of cell division and is often linked to more aggressive tumor behavior [9], which can be helpful for doctors in diagnosing cancer, determining its aggressiveness, and making treatment decisions. Research has indicated that elevated Ki-67 expression is linked to a higher tumor grade and a poor prognosis for individuals with breast cancer [10].

Ki-67 in breast cancer

Ki-67 has a connection to the cell cycle. It is observed during mitosis and in the mid-G1, S, and G2 phases signaling cell growth. In the G0 and early G1 phases, it is absent from silent or resting cells. This protein can be used as a marker to determine the developmental stage of a cell population because it is present in all replicating cells. Moreover, luminal-like breast tumors can be divided into luminal A and luminal B groups using the Ki-67. Luminal A tumors are estrogen receptor and progesterone receptor (ER/PR) positive and human epidermal growth factor receptor 2 (HER2/neu) negative with low Ki-67 protein. Luminal B tumors are estrogen receptor and progesterone receptor (ER/PR) positive and human epidermal growth factor receptor 2 (HER2/neu) variable with intermediate to high Ki-67 index [5]. Higher Ki-67 articulation has been associated with a high chance of relapse and a low prognosis in the early stage of breast cancer [11].

In a study on 2,573 instances of breast cancer, Liang et al. discovered an association between a high level of Ki-67 and a high histological grade [8]. Histological grade has a significant role in determining the course of breast carcinoma and can inform therapy choices [12]. Breast pathologists use the Nottingham change of the Scarff-Bloom-Richardson (SBR) review framework to determine histological grade. By examining the components of a tumor cell, such as tubule organization, nuclear pleomorphism, and mitotic count, grading is made. Consequently, tumor grading may be indirectly related to Ki-67 articulation [8]. The type of treatment selected is influenced by a number of additional prognostic factors, including the histological type of cancer, growth size, grade, age, estrogen receptor (ER) status, and the expansion marker Ki-67 [5]. Human epidermal growth factor receptor 2 (HER2/neu) articulation and hormone receptor status, including estrogen receptor (ER) and progesterone receptor (PR), are frequently utilized as indicators for breast sickness, which is examined using immunohistochemistry (IHC) [13].

The proliferative marker Ki-67 has been frequently utilized to predict the prognosis of breast cancer. Generally, high Ki-67 expression is linked to worse overall survival outcomes, including faster tumor growth, increased metastasis risk, and lower survival rates [14]. Ki-67 expression can help guide treatment decisions, particularly in selecting patients who may benefit from adjuvant chemotherapy [15]. This has been observed in various cancers such as breast, lung, and prostate [16]. In breast carcinoma, high Ki-67 levels are associated with a poor prognosis, which can influence treatment decisions such as chemotherapy [17]. Ki-67 expression can be heterogeneous within a tumor, and scoring methods can vary between laboratories [18]. Depending on the kind of cancer, different cutoff values for high Ki-67 expression may apply [19].

Although Ki-67 has been the subject of numerous published studies evaluating its predictive value in breast cancer, it is still not regarded as a clinically useful diagnostic. Understanding the relationship between Ki-67 and the established pathological parameters along with estrogen receptor (ER)/progesterone receptor (PR) and human epidermal growth factor receptor 2/neu proto-oncogene (HER2/neu) status is essential for developing treatment strategies and conducting clinical assessments [5].

## Materials and methods

The study was conducted in Bijapur Lingayat District Education (BLDE) (Deemed to be University), Shri BM Patil Medical College Hospital and Research Centre in Vijayapura. It was an institutional-based cross-sectional study. The study population consisted of 55 cases of mastectomy specimens of primary breast cancer obtained at the Histopathology Section of the Department of Pathology. The study was conducted between September 1, 2022, and April 30, 2024. The exclusion criteria included incomplete data and improperly fixed specimens along with biopsy specimens.

The specimens underwent standard processing after being preserved in 10% formalin. Sections with a thickness of 4 microns were cut from the majority of the appropriate tissue blocks. For the purpose of morphologic diagnosis (World Health Organization classification) and grading (modified Scarff-Bloom-Richardson system of cancer grading), one section was stained with hematoxylin and eosin. Another four sections were placed on slides coated with poly-L-lysine and subjected to estrogen receptor (ER)/progesterone receptor (PR), human epidermal growth factor receptor 2/neu (HER2/neu) proto-oncogene, and Ki-67 immunohistochemical staining.

We also reviewed the slides of histologically proven cases of breast carcinoma over the past year and retrieved the blocks for immunohistochemistry. Mouse monoclonal primary antibody against Ki-67 (DAKO, Glostrup, Denmark) was used for immunohistochemical study of Ki-67. The details of every case, including patient age, tumor site, tumor size, tumor type, histological grade, lymph node invasion, and vascular invasion were collected from original pathology reports. The immunohistochemical expression of Ki-67 was then correlated with these established prognostic factors along with estrogen receptor (ER)/progesterone receptor (PR) and human epidermal growth factor receptor 2/neu (HER2/neu) proto-oncogene status and added into a data entry form. The data was then statistically analyzed.

Assessment of Ki-67

Ki-67 expression is based on nuclear expression by tumor cells. A minimum of 1,000 cells were counted in each of 10 high-power fields to determine the proportion of Ki-67-positive tumor cells. The score was based only on tumor cells that were nuclear-positive [5].

Ki-67 labeling index ​​​(LI) is defined as the ratio of positively stained tumor cells to the total number of malignant cells counted [5]: Ki-67 labeling index = (number of cells showing positive nuclear staining / total number of cells) × 100.

Grading of Ki-67 expression

Ki-67-expressing tumors were categorized into two groups: low proliferative (Ki-67 is positive in less than 20% of tumor cells) and high proliferative (Ki-67 is positive in at least 20% of tumor cells) [5].

Ki-67 is primarily a nuclear marker; hence, occasional membrane and cytoplasmic staining of Ki-67 across specimens should be ignored while using the mind bomb E3 ubiquitin protein ligase 1 (MIB1) antibody. Internal positive controls, such as lymphocytes, mitotic figures, normal ductal epithelial cells, and endothelial and stromal cells, are helpful. In some mastectomy specimens, biological heterogeneity of Ki-67 staining can occur. In such a scenario, scoring should be done on tumor edges or hot spots [20].

Interpretation of estrogen receptor (ER)/progesterone receptor (PR) and human epidermal growth factor receptor 2/neu (HER2/neu) proto-oncogene status was done according to guidelines provided by the American Society of Clinical Oncology/College of American Pathologists (ASCO/CAP). Estrogen receptor (ER)/progesterone receptor (PR) status was evaluated based on nuclear expression. Human epidermal growth factor receptor 2/neu (HER2/neu) proto-oncogene status was interpreted based on patterns of membranous staining in tumor cells.

Ethical clearance and consent

Approval was taken from the Institutional Ethics Committee of Bijapur Lingayat District Education (BLDE) (Deemed to be University) Shri BM Patil Medical College Hospital and Research Centre before study commencement with approval number BLDE(DU)/IEC/681/2022-2023. Informed and written consent was taken from all the study participants.

Statistical analysis

The collected data was analyzed using Microsoft Excel spreadsheet (Microsoft Corp., Redmond, WA) and SPSS version 20 (IBM SPSS Statistics, Armonk, NY). Findings were displayed as counts, percentages, and mean±standard deviation (SD). The chi-square test was used to compare categorical variables. Statistics are taken as significant when p<0.05. A two-tailed statistical test was done.

## Results

As shown in Table 1, out of 55 breast cancer cases, a slightly higher proportion of cases (31 cases) corresponding to 56.4% are in individuals older than 50 years. Most tumors are less than 2 cm, indicating early detection or smaller tumor sizes being more common. Vascular invasion is absent in 32 cases corresponding to 58.2%, suggesting that many tumors have not yet invaded the blood vessels, which could be a favorable prognostic indicator. Most tumors are of grade II (intermediate grade), followed by grade I (low grade), and grade III (high grade) malignancies. The majority of cases are estrogen receptor (ER)-negative, which might indicate a more aggressive tumor type and potential resistance to hormone therapy. A higher proportion of cases are progesterone receptor (PR)-negative, which could also influence treatment options and prognosis. Human epidermal growth factor receptor 2/neu (HER2/neu) positivity is present in nearly half of the cases as indicated in Table 1.

**Table 1:** Immunopathological and clinicopathological characteristics of 55 patients with breast cancer ER: estrogen receptor, PR: progesterone receptor, HER2/neu: human epidermal growth factor receptor 2/neu

Tumor variables		Number of cases	Percentage (%)
Age	<50 years	24	43.6
	>50 years	31	56.4
Tumor size	<2 cm	36	65.5
	2-5 cm	11	20
	>5 cm	8	14.5
Lymph node status	Involved	37	67.3
	Not involved	18	32.7
Vascular invasion	Absent	32	58.2
	Present	23	41.8
Histological grade	I	17	30.9
	II	26	47.3
	III	12	21.8
ER status	Positive	16	29.1
	Negative	39	70.9
PR status	Positive	24	43.6
	Negative	31	56.4
HER2/neu	Positive	26	47.3
	Negative	29	52.7
Ki-67	High grade	30	54.5
	Low grade	25	45.5

**Figure 1:**
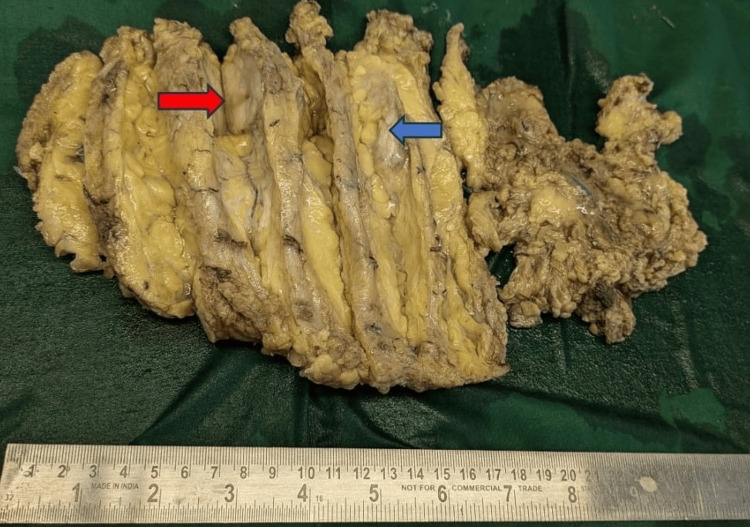
Larger tumor (red arrow) measuring 2×1×1 cm seen in the upper inner quadrant and smaller tumor (blue arrow) measuring 1.5×1×0.5 cm situated in the upper outer quadrant in a right mastectomy specimen

Figure 1 shows a mastectomy specimen we received in our histopathology section measuring 10×4×2 cm. Two tumors were noted with a larger tumor (red arrow) measuring 2×1×1 cm and a smaller tumor (blue arrow) measuring 1.5×1×0.5 cm.

**Figure 2:**
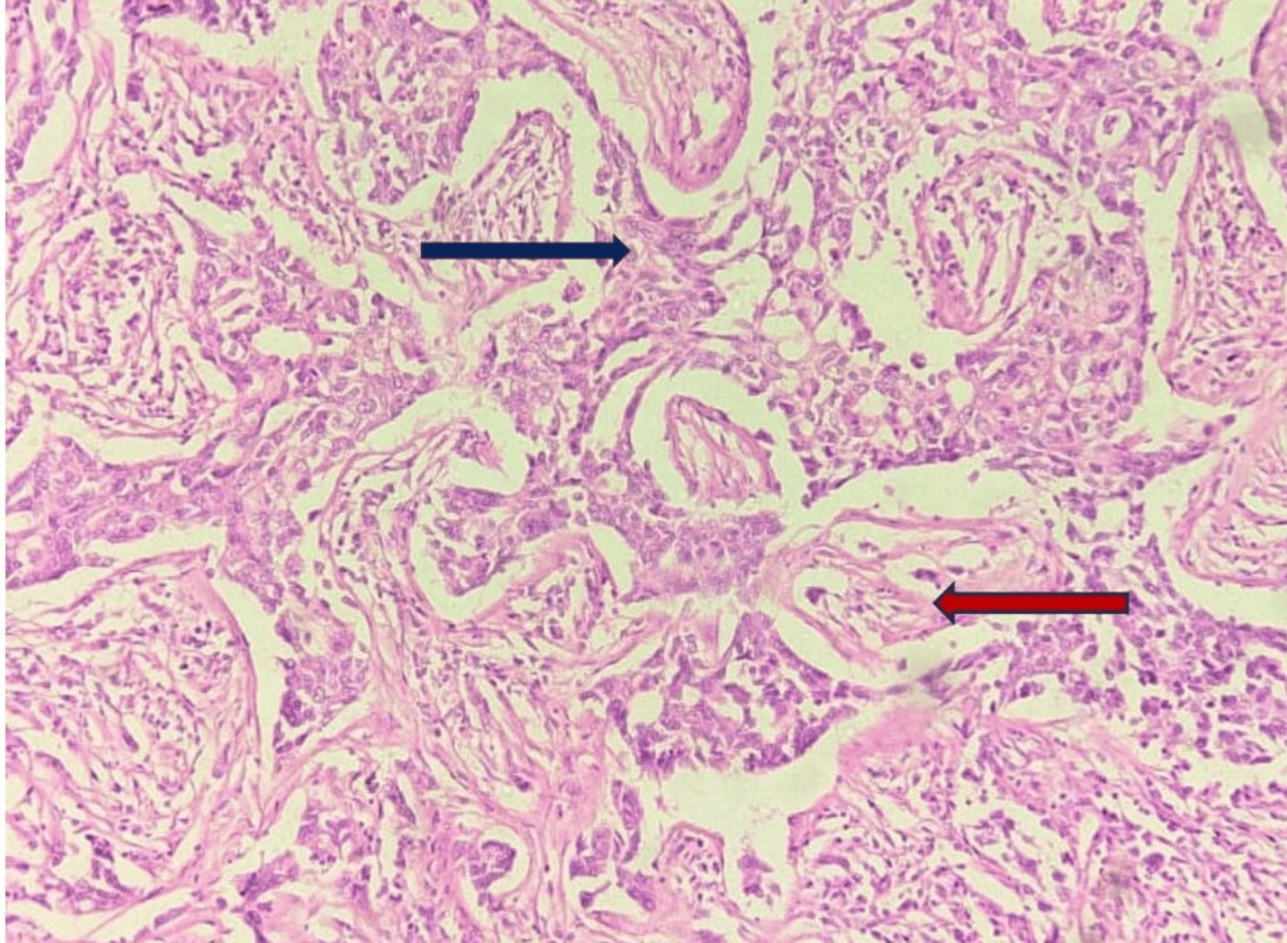
H&E-stained slide of tumor tissue of IBC seen arranged in tubules (navy blue arrow) showing moderate pleomorphism separated by fibro-collagenous septa (red arrow) with no mitotic figures noted in this field (20×) H&E: hematoxylin and eosin, IBC: invasive breast cancer

Hematoxylin and eosin-stained slides for every case were assessed histologically, and a score was given based on tubule formation, nuclear pleomorphism, and mitotic count (modified Scarff-Bloom-Richardson grading). The tumor was graded as indicated in Figure 2.

**Figure 3:**
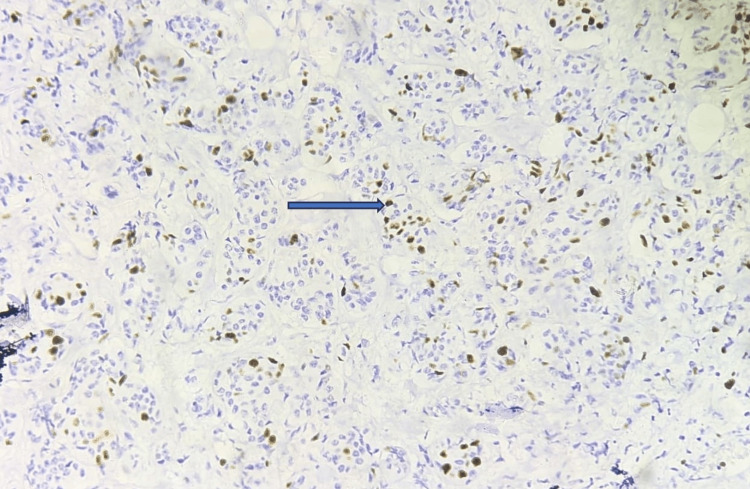
Expression of Ki-67 staining in IBC Ki-67 protein is found to be expressed in the nucleus of breast cancer cells (blue arrow). A high-grade expression of 20%-24% was found in this case (IHC stain, 20×). IBC: invasive breast cancer, IHC: immunohistochemistry

Analyses of the staining status, pattern, and intensity of Ki-67 expression were performed in malignant tumor cells. All positive cases showed nuclear positivity. Ki-67 expression was evaluated by quantity score. In our study, high-grade expression of Ki-67 was seen in 30 cases out of 55 cases constituting 54.5%. The expression of Ki-67 staining is found to be expressed on the nucleus of breast cancer cells as indicated in Figure 3 (blue arrow). A high-grade expression indicates tumor aggressiveness predicting a poor prognosis in breast cancer patients.

**Figure 4:**
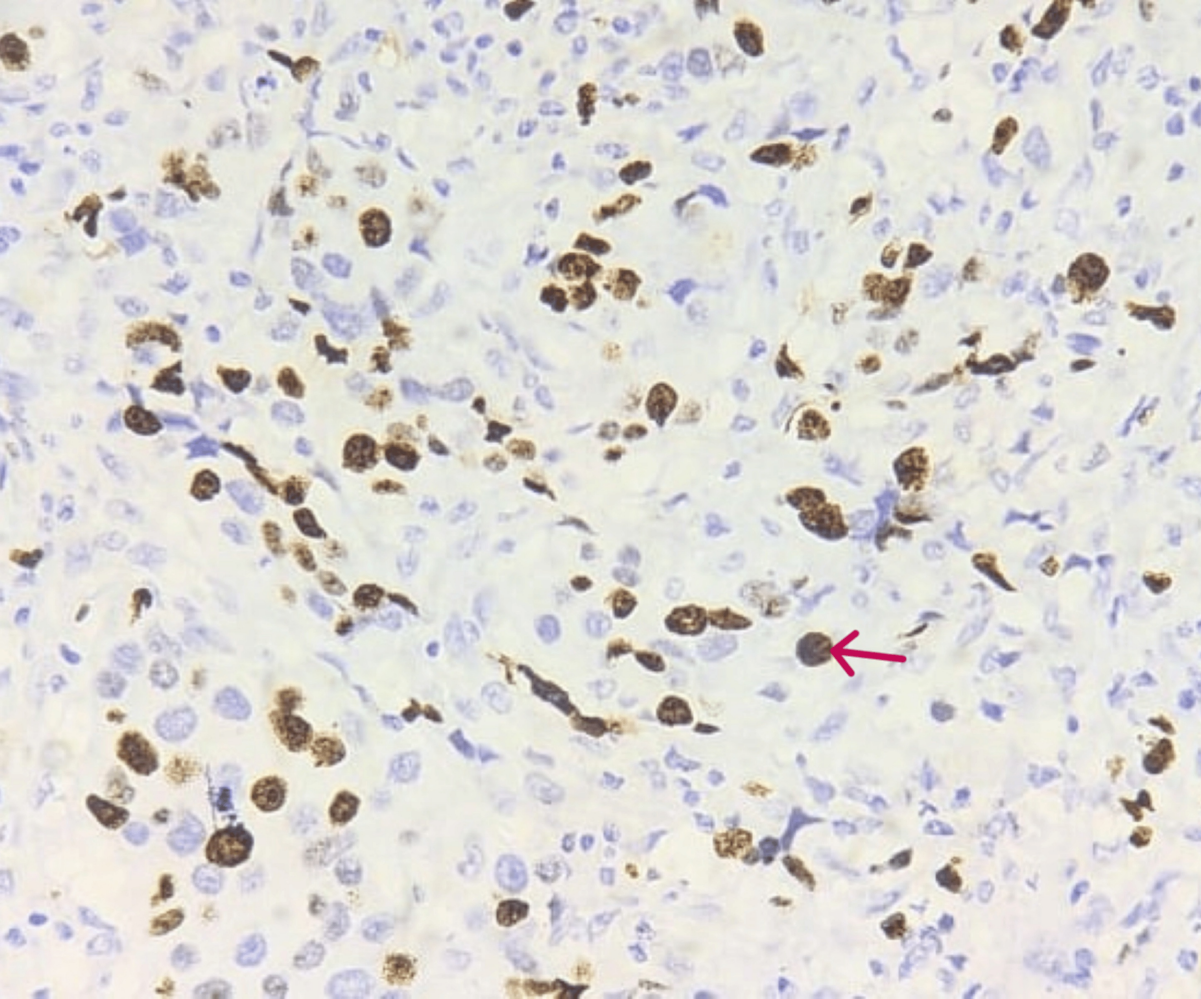
IHC-stained slide showing the expression of Ki-67 in IBC Ki-67 is strongly expressed in the nucleus of tumor cells (red arrow) showing a high grade of 28%-32% indicating aggressiveness of the tumor (40×). IBC: invasive breast cancer, IHC: immunohistochemistry

A high-grade Ki-67 labeling index of 28%-32% was found to be expressed indicating a poor prognosis. Ki-67 is a nuclear protein expressed in the nucleus of tumor cells as indicated by the red arrow in Figure 4 (under 40×).

**Figure 5:**
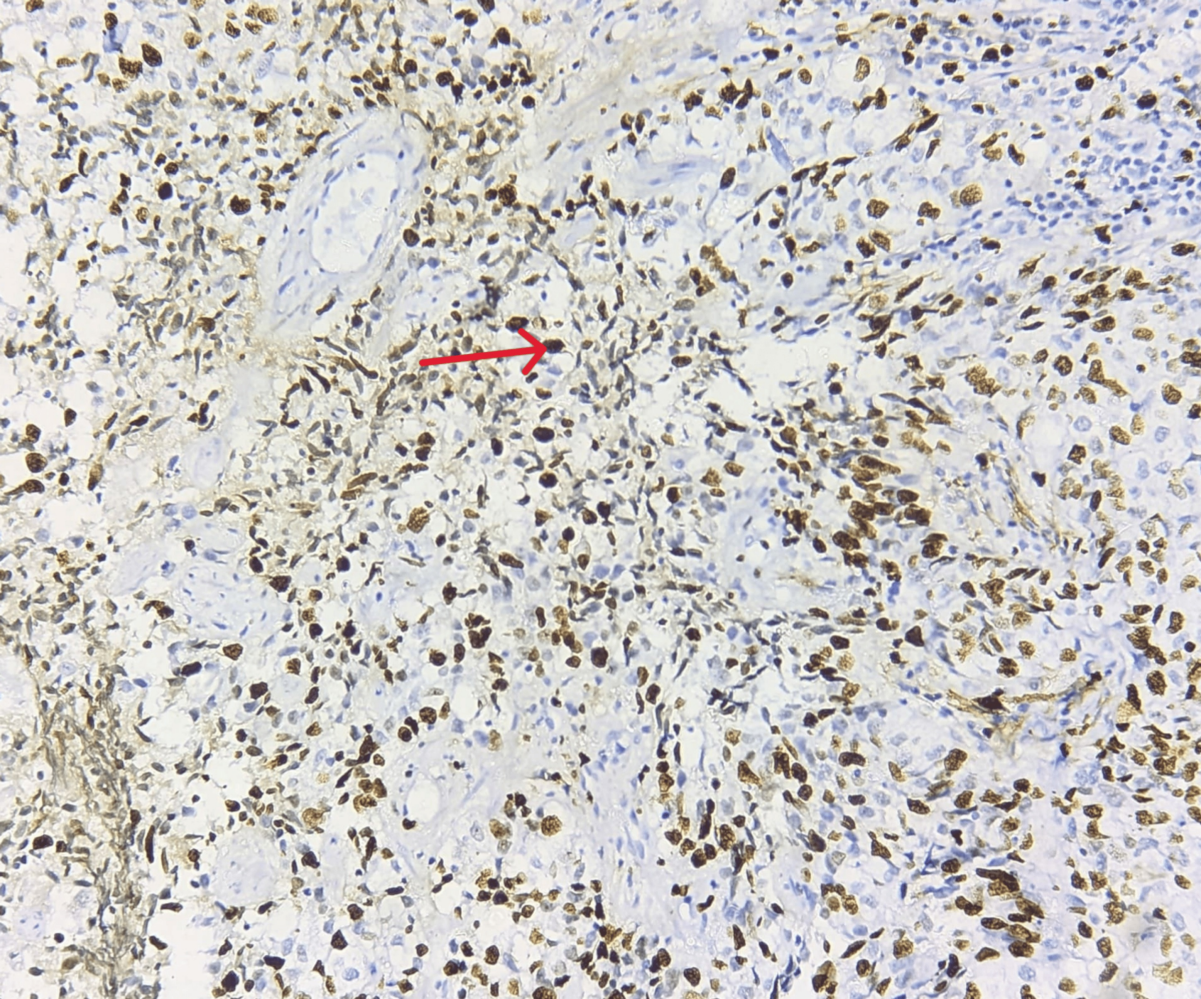
Strong expression of Ki-67 in IBC Ki-67 a nuclear protein showing a proliferative index of 34%-35%, a very high-grade Ki-67 expression (red arrow), indicating a very poor prognosis (IHC stain, 40×). IBC: invasive breast cancer, IHC: immunohistochemistry

A strong expression of Ki-67 with a proliferative index of 34%-35% indicates a very poor prognosis as shown in Figure 5 (under 40×).

**Figure 6:**
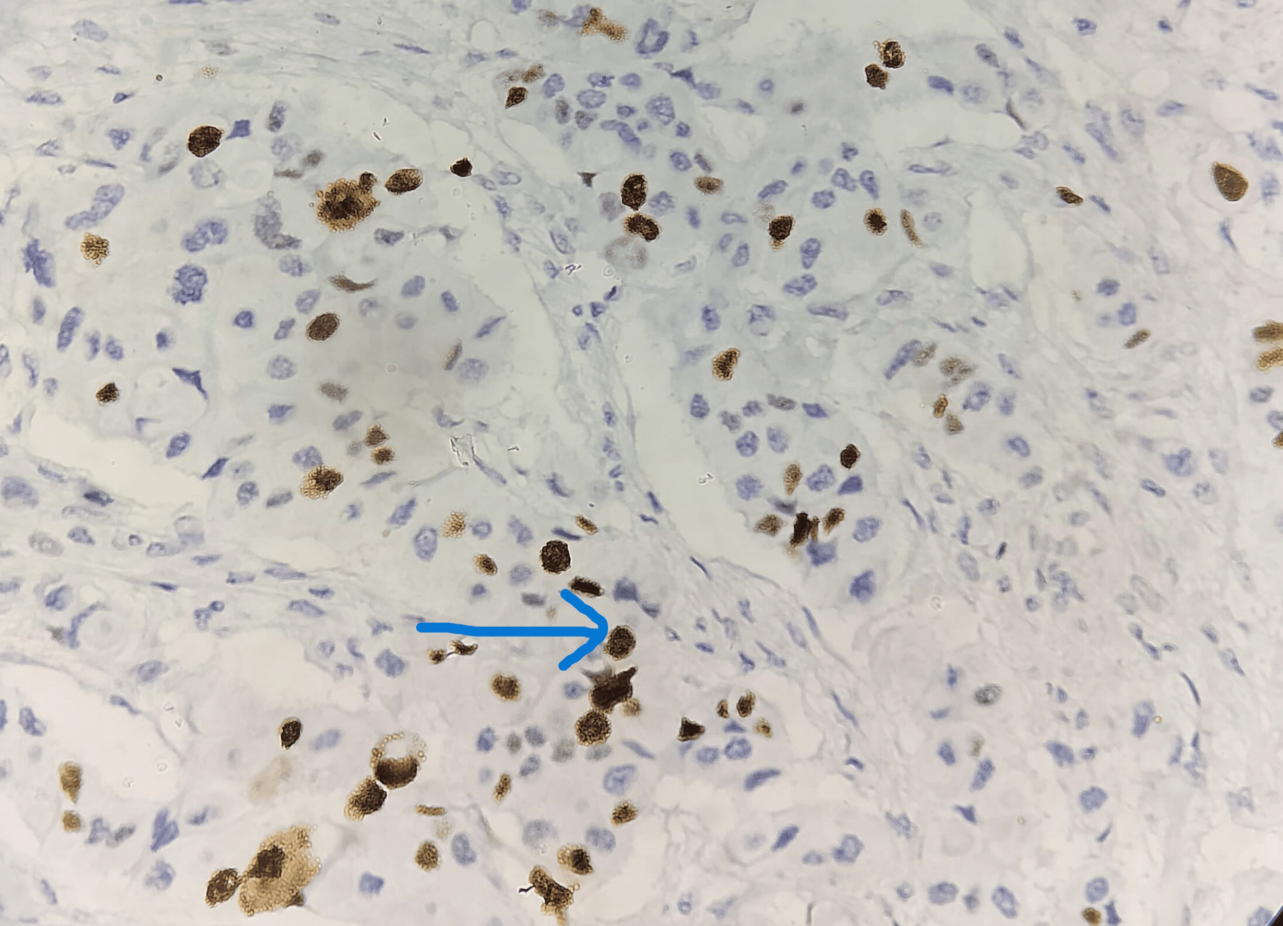
Ki-67 staining in IBC showing nuclear expression of low grade (15%-18%) indicated by the blue arrow (IHC stain, 40×) IBC: invasive breast cancer, IHC: immunohistochemistry

**Figure 7:**
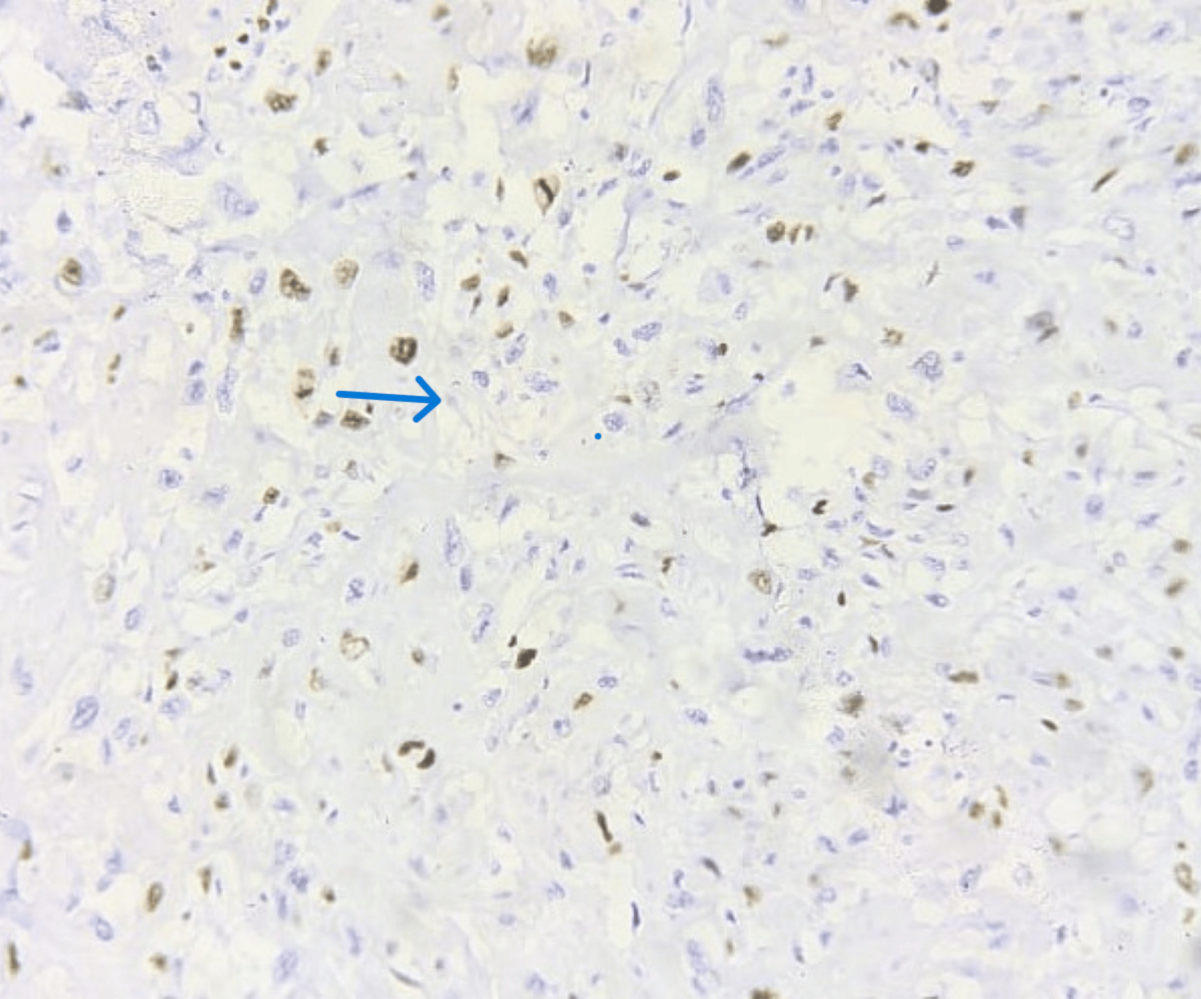
Ki-67 staining in IBC showing nuclear expression of low grade (7%-9%) indicated by the blue arrow (IHC stain, 20×) IBC: invasive breast cancer, IHC: immunohistochemistry

Ki-67 with low-grade expression was seen in the remaining 25 cases constituting 45.5% A low Ki-67 labeling index of less than 20% was considered low grade as indicated in Figure 6 and Figure 7.

Table 2 provides an analysis of the labeling index (LI) for Ki-67 in breast cancer cases, separated into two groups: those with a labeling index of less than 20% and those with a labeling index of more than 20%. Of the 55 cases of breast cancer, 25 (45.5%) cases showed low Ki-67 proliferative activity as shown in Figure 7. The remaining 30 (54.5%) cases had high Ki-67 labeling index (Ki-67 LI > 20%) as indicated in Figure 5. Tumor size and histological grade are significantly associated with Ki-67 expression. High Ki-67 expression is seen to be associated with larger tumors (p<0.05) and high-grade tumors (p<0.001). There was no discernible association between Ki-67 and other histopathological features such as age, vascular invasion, estrogen receptor (ER), progesterone receptor (PR), human epidermal growth factor receptor 2/neu (HER2/neu), and lymph node status. These results imply that tumor size and histological grade are strongly correlated with Ki-67 expression, indicating its potential role in tumor aggressiveness and prognosis.

**Table 2:** Expression of Ki-67 in breast cancer and its association with clinicopathological factors ER: estrogen receptor, PR: progesterone receptor, HER2/neu: human epidermal growth factor receptor 2/neu

Tumor variables		Ki-67 LI < 20%		Ki-67 LI > 20%		P-value
		Number	%	Number	%	
Age	<50 years	11	20	13	23.6	>0.05
	>50 years	14	25.5	17	30.9	-
Tumor size	<2 cm	16	29	20	36.4	<0.05
	2-5 cm	7	12.7	4	7.3	-
	>5 cm	2	3.6	6	10.9	-
Lymph node status	Involved	14	25.5	23	41.8	>0.05
	Not involved	11	20	7	12.7	-
Vascular invasion	Absent	17	30.9	15	27.3	>0.05
	Present	8	14.5	15	27.3	-
Histological grade	I	17	30.9	0	0	<0.001
	II	6	10.9	20	36.4	-
	III	2	3.6	10	18.2	-
ER status	Positive	6	10.9	10	18.2	>0.05
	Negative	19	34.5	20	36.4	-
PR status	Positive	11	20	13	23.6	>0.05
	Negative	14	25.5	17	30.9	-
HER2/neu	Positive	12	21.8	14	25.5	>0.05
	Negative	13	23.6	16	29.1	-

## Discussion

We have studied Ki-67 expression along with routinely used markers estrogen receptor (ER), progesterone receptor (PR), and human epidermal growth factor receptor 2/neu (HER2/neu) in a total number of 55 cases. The findings of Ki-67 were correlated with histopathological parameters such as tumor size, tumor grade, vascular invasion, lymph node metastases, age, and site of the tumor, as well as estrogen receptor (ER), progesterone receptor (PR), and human epidermal growth factor receptor 2/neu (HER2/neu) status.

The majority of cases in our study showed a statistically significant correlation between Ki-67 and tumor size. A total of 20 out of 30 cases or 66.7% of cases with high-grade Ki-67 expression belonged to the size range of 2-5 cm and had a p-value of less than 0.05. According to this, Ki-67 expression may be related to tumor growth, which is in line with the research of Nishimura et al. [21]. Nishimura et al. in their study reported the mean tumor size with high-grade Ki-67 expression as 2.7 cm with a standard deviation of 2.0 and a p-value of less than 0.0001 [21]. As shown in Table 3, Kamranzadeh et al. also reported a p-value of 0.04 with 15 (45.45%) cases of low-grade Ki-67 expression and 45 (60.81%) cases of high-grade Ki-67 expression belonging to tumor size 2-5 cm reporting highly significant results [22]. Our findings were also similar to the study findings of Lombardi et al., where they found that the mean tumor size with high-grade Ki-67 expression was 1.82 cm with a standard deviation of 10.4 and a p-value of 0.0001 [23]. No statistically significant correlation was seen between Ki-67 (p=0.94) and tumor size (p=0.7) in the investigations conducted by Gogoi et al. [5] and Soliman et al. [20]. Gogoi et al., in their study, found that the majority of cases, i.e., 22 out of 34 cases, with high-grade Ki-67 expression belonged to tumor size 2-5 cm without any statistically significant association [5].

**Table 3:** Association between tumor size and Ki-67 (comparison of statistical results of different studies)

Author	P-value for comparison of Ki-67 with tumor size	Statistical significance
Present study	<0.05	Significant
Gogoi et al. (2021) [5]	0.94	Not significant
Soliman et al. (2016) [20]	0.7	Not significant
Nishimura et al. (2010) [21]	<0.0001	Highly significant
Kamranzadeh et al. (2019) [22]	0.04	Significant
Lombardi et al. (2021) [23]	<0.0001	Highly significant

In the current research, the majority of cases with high Ki-67 expression, i.e., 20 out of 30 cases corresponding to 66.7%, belonged to grade II, and the rest (10 cases) constituting 33.3% of cases belonged to grade III with a p-value of <0.001, reflecting a very high significant association between Ki-67 and the modified Scarff-Bloom-Richardson grading. This result was consistent with the findings of Nishimura et al. [21] and Lombardi et al. [23], with observed p-values of less than 0.0001 as shown in Table 4. Similarly, Soliman et al. [20] and Gogoi et al. [5] found a highly significant correlation, with a p-value of 0.005. Gogoi et al. found in their study that 86% of cases, i.e., 12 cases, under histological grade III expressed high Ki-67, while 11 cases, constituting 92% of cases, under grade I had Ki-67 expression of <20% [5]. Our study finding was discordant with the findings of Kamranzadeh et al., where 63 cases of high Ki-67 expression corresponding to 85.2% of cases were categorized under tumor grade I and II combined with no statistical significance between Ki-67 and tumor grade as shown in Table 4 [22]. This discrepancy may be attributed to the difference in the cutoff range for categorizing low and high expression of Ki-67. Kamrazadeh et al. had taken 10% as the cutoff, but in our study, similar to other studies, we have taken 20% as the cutoff.

**Table 4:** Association between Ki-67 and the modified Scarff-Bloom-Richardson grading (comparison of statistical results of different studies)

Author	P-value for comparison of Ki-67 with the Scarff-Bloom-Richardson grading system	Statistical significance
Present study	<0.001	Highly significant
Gogoi et al. (2021) [5]	0.002	Highly significant
Soliman et al. (2016) [20]	0.005	Highly significant
Nishimura et al. (2010) [21]	<0.0001	Highly significant
Kamranzadeh et al. (2019) [22]	0.19	Not significant
Lombardi et al. (2021) [23]	<0.0001	Highly significant

Limitations of the study

In our study, a smaller sample size was taken resulting from the high cost of markers utilized. Since Ki-67 has shown a statistically insignificant relationship with many of the established pathological parameters, a study with a larger sample size is needed. Further multicentric studies will definitely provide a better insight into its correlation with the known clinicopathological parameters.

Future recommendations

To properly ascertain the usefulness of this biomarker for use as a diagnostic and prognostic tool in routine cases of breast cancer, more studies are required with a much bigger sample size with interlaboratory standardization in relation to multiple additional factors that will help in the treatment of human breast cancer.

## Conclusions

A cross-sectional study was performed on 55 cases of mastectomy specimens diagnosed with invasive breast carcinomas to study the immunohistochemical expression of Ki-67 in tumor cells. The expression of Ki-67 was correlated with various prognostic parameters such as patient age, tumor size, histological grade, lymph node status, tumor stage, and ER, PR, and HER2 status. However, the expression of Ki-67 was statistically significant with histological grading and tumor size in our study. Immunohistochemical interpretation of Ki-67 proliferation index should be conducted in routine cases of breast carcinoma to obtain clinically useful information on tumor aggressiveness and treatment decision. Hence, Ki-67 may be used as a predictive and prognostic marker in managing breast cancer patients.
